# Lipid apheresis techniques: current status in Germany

**DOI:** 10.1007/s11789-012-0047-5

**Published:** 2012-05-15

**Authors:** Peter Grützmacher, Claudius Kleinert

**Affiliations:** Medizinische Klinik 2, Markus Krankenhaus, Frankfurt/Main, Germany

**Keywords:** Lipid apheresis, Hypercholesterolemia, Lipoprotein a, LDL cholesterol

## Abstract

For long-term lipid apheresis therapy, several different technical systems have been developed which enable effective reduction of LDL cholesterol and other atherogenic lipoproteins, such as Lp(a), with sufficient selectivity and good clinical tolerance. Suitable techniques include whole blood adsorption with polyacrylamide and dextran sulfate cellulose, while primary plasma separation is used for cascade filtration, heparin-induced precipitation, immunoadsorption, silicate gel adsorption, and dextran sulfate cellulose (both techniques).

The technical features are described. Only intensive training and experience of the medical personnel guarantees reliable treatment safety of all systems at a very low rate of minor side effects.

## Introduction

Over the past 3 decades, the lipid apheresis techniques have progressed considerably. At present, 6 different systems are available which have been approved by the technical surveillance institute—called TÜV—and been awarded a so-called CE-sign. All systems now have fully automated process management. A continuous manual steering is no longer necessary.

All methods fulfill the current legal requirements for LDL apheresis, made by the German Gemeinsamer Bundesausschuss (GBA). More than 60% LDL reduction has to be achieved per apheresis session within 6 h.

This report focuses on technical aspects of the lipid apheresis technique currently used in Germany.

Although the technical principles of lipid apheresis are basically different, the requirements are easily fulfilled by all techniques, usually within a 1.5–3 h session. [For review see^3^,^7^,^8^,^11^,^14^,^15^].

The lipid reduction rates of the different techniques are shown in Table 1.

**Table 1:** Reduction rates of serum (lipo)proteins with current LDL apheresis systems (according to literature). (Modified from [4])

	Lipid filtration	HELP	DALI	DSA	LA
LDL cholesterol (%)	61–68	55–61	53–76	49–75	62–69
HDL cholesterol (%)	6–17	5–17	05–29	4–17	9– 27
Lp(a) (%)	61–74	55–68	28–74	19–70	51–71
Triglycerides (%)	38–56	20–53	29–40	26–60	34–49
Fibrinogen (%)	42–51	51–58	13–16	17–40	15–21
IgG (%)	14	16	15–20	11	25

## Whole blood apheresis systems

The progress in adsorber technology has given rise to a considerable simplification of purification techniques. So far, only the adsorption technology is suitable for whole blood apheresis. Adsorbing substances in microporous granula of larger diameters allows the blood to flow through the adsorber without clotting. Heparin and citrate are used for anticoagulation. The buildup is simple; 1.5–2 blood volumes are usually processed.

The principle is also called hemoperfusion, well-known in the treatment of severe intoxication.

**1. Polyacrylamide** **-** **adsorption**

With the DALI system (abbreviation for*d*irect*a*dsorption of*li*poproteins), negatively charged polyacrylamide is fixed on Eupergit^®^ beads, binding to the positively charged APO B 100 region of LDL, VLDL, and Lp(a) particles. Adsorber sizes range from 300–1,200 ml, manufactured for single use. Citrate is used for anticoagulation. ACE-inhibitor treatment is contraindicated [1,16].

**2. Dextran sulfate cellulose adsorption**

More than 20 years before its introduction as a whole blood adsorption technique, dextran sulfate cellulose was (and is still) used for LDL apheresis of plasma. Negatively charged dextran sulfate is fixed on cellulose beads, which is the ligand for the abovementioned lipoproteins.

Different adsorber capacities are available for single use (DL 50, DL 75, DL 100). Citrate is used for anticoagulation; combination with heparin is possible. The use of ACE inhibitors is contraindicated because the membrane generates bradykinine, a potent endogenous vasodilatator which cannot be metabolized when ACE inhibitors are given, resulting in serious hypotensive and anaphylactoid reactions.

## Systems with plasma separation

Since the early days of LDL apheresis, the plasma separation has been used to isolate plasma from blood cells. For plasma separation, hollow capillary fiber filters or centrifuge systems are used. Successful treatment has been described in some patients treated using simple plasma separation for more than 6–9 years, replacing plasma with nearly iso oncotic albumin (5%) derived from blood donors [13].

Processing the patient’s plasma by adsorption, precipitation or a second filtration permits reinfusion of the patient’s own plasma, cleared from atherogenic lipoproteins. Within 2–3 h, 1–1.5 plasma volumes are usually processed [2].

**1. Cascade filtration**

This technique requires primary plasma separation; usually a hollow fiber filter is used to separate plasma from blood cells. Plasma is processed through a second hollow fiber filter, permeable for particles with a molecular weight below 50,000–100,000 Da, allowing HDL, albumin, and smaller immunoglobulins to pass, whereas LDL, Lp(a), VLDL and chylomicrones, as well as larger immunoglobulins like IgM, are retained. In contrast to the first decade of lipid apheresis treatment with this technique, improvement in membrane technology has made the loss of HDL and serum proteins, especially serum albumin, negligible. The term lipid filtration was proposed for the use of highly selective filters (EC_50_ W, Diamed company).

Fully automated apheresis machines have been developed so that continuous manual steering of flows and pressures is no longer required (Octo Nova/Diamed, Monet/Fresenius, Germany).

Warming of plasma to 38.5°C improves the sieving characteristics of larger lipoproteins compared to HDL. Heparin and citrate or both can be used for anticoagulation. The cascade filters are manufactured for single use [4,5].

**2.** ***H*** **eparin** **-** **induced** ***e*** **xtracorporeal** ***L*** **DL** ***p*** **recipitation (HELP system)**

The HELP system was developed in Germany in the 1980s, using the laboratory technique of determining HDL cholesterol by precipitation of LDL and VLDL at low pH in the presence of unfractionated heparin. After plasma separation, the plasma is diluted 1:1 with 0.2M sodium acetate buffer (pH 4.85), containing 100,000 IU heparin/L. At a low pH of 5.12, a heparin-protein complex, consisting of LDL, Lp(a), fibrinogen and VLDL, precipitates, which is cleared by a polycarbonate precipitation filter; thereafter, heparin is absorbed by a DEAE-cellulose-adsorber. Finally, the low pH plasma is buffered by a single path bicarbonate hemodiafiltration. For anticoagulation of the blood circulation, fractionated and unfractionated heparin can be used. The size of precipitation filter and adsorber has been increased, now enabling treatment of up to 4 L of plasma.

Despite the complex procedure, a considerable simplification and shortening of treatment has been achieved by premanufactured devices and fully automated rinsing processes.

The pronounced depletion of fibrinogen (also in cascade filtration) is assumed to have a contributory antiatherogenic potential [9,10,12].

**3. Dextran sulfate cellulose adsorption**

This technique was introduced in Japan in the early 1980s. The adsorption principle has been described above. The system is designed for single use. Adsorption of HDL cholesterol (which has no negatively charged domain) is negligible. A double column system has been developed, switching after every 500 ml of treated plasma volume to the other column, simultaneously flushing the lipoprotein-saturated column with a phosphate buffer for regeneration. Theoretically, the technique permits treatment of an unlimited amount of plasma, which is not possible with whole blood adsorption techniques. Heparin is used for anticoagulation [14,15].

**4. Silicate gel adsorption**

Recently a plasma adsorption technique using silicate gel granula was introduced. Microporous silicate beads, negatively charged on the surface, bind to the positively charged APO B region of LDL and VLDL, similar to dextran sulfate and polyacrylamide, leaving HDL particles unaffected.

Heparin is used for anticoagulation.

The Adasorb^®^ machine (Medicap Company) is used for plasma processing. For primary plasma separation, several primary units are applicable, such as Cobe Spectra^®^, Life18^®^, requiring the combination of two apheresis units of different distributors.

A double column technique is used, changing the absorber during treatment, with simultaneous regeneration of the saturated adsorber. As a technique for regeneration and disinfection has now been developed, the system can be reused for more than 30 treatments (lipo collect 300^®^, Medocon company, Germany).

**5. Immunoadsorption**

Polyclonal antibodies against human APO B100, derived from sheep, are fixed on Sepharose CL-4B, which bind all APO B100-containing lipoproteins (LDL, Lp(a), VLDL) specifically. However, there is considerable nonspecific binding of other plasma proteins.

A double column system is used, changing to the second column after processing of every 500 ml of plasma, simultaneously flushing the saturated column with acid glycine for regeneration.

Production of the columns is expensive. Therefore, the system must be reused for 20–40 sessions per column, two column sizes are available. Heparin and citrates or both can be used for anticoagulation.

After the end of the session, the regenerated columns are filled with antibacterial sodium acid and stored at 4°C. Careful flushing with isotonic saline is necessary before the next treatment [3,15].

## Further specific indications

For*isolated hyperlipoproteinemia* (*a*), all systems are effective because the behavior of Lp(a) is similar to LDL, which is also part of the Lp(a) particle.

Efficient elimination of phytanic acid, which is elevated in the rare*Refsum’s syndrome*, has been described by several systems, such as DALI, HELP, and cascade filtration. Elimination is easily possible because phytanic acid in plasma is largely bound to LDL and VLDL particles [3].

The*chylomicronemia syndrome* is characterized by a massive increase of triglyceride-rich plasma lipids. Triglyceride levels exceeding 5,000–10,000 mg/dL can be observed. The plasma lipid concentration is 10–20 fold higher than in LDL apheresis patients.

Absorption techniques are regularly not effective enough due to rapid saturation of the current adsorber. The cascade filtration or even pure plasma exchange is superior in these cases.

## Safety of apheresis techniques

The observation of side effects in clinical studies is usually based on very low number of patients and treatments. Much more experience has been gathered by monitoring the routine treatment, initiated by the manufacturers in cooperation with apheresis specialists [for review see^3^–^5^,^7^,^9^,^11^,^14^–^16^, and Table 2].

**Table 2:** Common side effects of lipid apheresis treatment [1,3–7,9,11,12,15,16]

Method	Treatment records	Total incidence (%)	Frequent/less severe side effects	Associated symptoms
HELP	75,061	3.05	Hypotension, angina, headache, nausea, weariness, edema, eye pressure	↓ Coagulation
Cascade-filtration	1,708	2	Hypotension, fatigue, edema	Protein loss (not with EC_50_)
DALI	12,291	3.85	Hypotension, nausea, vomiting, chest pain, flush	↑ Bradykinine
Dextran sulfate adsorption	Not reported	0.3–0.9	Hypotension, paresthesias, pain, nausea, vertigo	↑ Bradykinine, ↓ coagulation
Immunoadsorption	2,600	< 2	Hypotension, nausea, vertigo	Antibodies (sheep), reuse

Serious complications are rare and severe, ranging < 0.1‰–< 1%, usually causing hospital admission

Allergic reaction 0.25%, fever 0.2%, hemolysis 0.05%, dyspnea 0.1%, shock 0.2%, arrhythmia 0.04%

In general, the lipid apheresis is well tolerated and procedures are safe. Lethal events have not been published.

Serious complications are rare, ranging from < 0.1‰–< 1%. Allergic reactions, fever, dyspnea, cardiac arrhythmias, hemolysis, and shock have been documented as rare events.

Although blood coagulation is deeply disturbed for several hours after apheresis, more or less by all systems, bleeding complications have not been reported [15].

In general, the patients should be instructed to report any changes in medication immediately before the next treatment sessions since an unexpected risk may occur if the medication is changed, e.g. by cardiologists or general practitioners, introducing ACE-inhibitors which are contraindicated for most adsorption techniques (see above). Here the use of renin inhibitors and AT1 antagonists is recommended.

Mild hypotension occurs occasionally, usually resolved after the end of treatment, and may be accompanied by nausea, vertigo, fatigue, mild headache and vomiting. This mild cardiocirculatory instability is mostly induced by the extension of plasma volume to the extracorporeal circuit; moderate loss of serum proteins may aggravate symptoms and can lead to mild edema when higher volumes of saline infusions are used. However, in all currently used systems a minor loss of proteins no longer requires albumin substitution and is no longer of clinical relevance.

A nonspecific loss of γ-globulins in the range of 5–10% occurs, but there is no evidence of increased susceptibility to infections. In routine practice, an apheresis session is often scheduled some days later in the patients with active infections.

In patients with immunoadsorption, the sheep antibodies to human APO B can be detected, not inducing any clinical diseases.

An additional filter behind the adsorber is used in some plasma apheresis systems to enhance protection from unwanted contamination of reinfused patient plasma with microparticles from the adsorber; however, this is not possible when whole blood adsorption systems are applied.

Meanwhile, all the systems have fully automated process monitoring, which protects from most procedural complications. However, the technical principles are complicated and require a thorough understanding of the underlying physical processes.

Intensive training of medical staff including physicians is mandatory to maintain treatment safety since none of the systems offers protection from malpractice through inexperienced staff.

For ambulatory treatment, a special experience with extracorporeal therapy and dialysis, as well as a specialist medical qualification in nephrology, is required by German law.

In clinical settings, some apheresis procedures are often performed by bloodbanks, which have highly experienced and qualified staff for the processing of blood and plasma products.

### Conflict of interests

The author has accepted fees from the companies B. Braun and Fresenius, Germany. This article is part of a supplement sponsored by an unrestricted educational grant from B. Braun and Fresenius Medical Care.
